# Editorial Notes and Comments

**Published:** 1899-07

**Authors:** 


### *EDITORIAL NOTES AND COMMENTS.*

The Business office of THE JOURNAL-RECORD is 305, 306 Fitten Building.

The Editorial office is 318-319-320 The Prudential.

Address all Business communications to Dr. M. B. Hutchins, Mgr.

Make remittances payable to THE ATLANTA JOURNAL-RECORD OF MEDICINE.

On matters pertaining to the Editorial and Original Communications address Dr. Bernard Wolff, Atlanta.

Reprints of original articles will be furnished at cost price. Requests for the same should always be made *on the manuscript*.

We will present, post-paid, on request, to each contributor of an original article, twenty (20) marked copies of THE JOURNAL-RECORD containing such article.

THE Fiftieth Annual Meeting of the American Medical Association was held in Columbus, Ohio, on June 6, 7, 8, 9, 10. The attendance was large and the meeting proved to be one of great satisfaction. The association was welcomed by both the governor of the State and the mayor of the city, and the former in giving utterance to the fact that he hoped the "custodial institutions and hospitals might be kept far beyond political influence," was most vociferously cheered.

The president of the meeting, Dr. Jos. M. Matthews, of Louisville, Ky., delivered an annual address, which was largely in the manner of suggestions as he thought of needed reforms. Among these was the fact that the association needed a permanent home and suggested Washington, D. C., as the place most suitable, giving many cogent reasons. To this we heartily agree, for there are many reasons why the home of a national association should be at the national capital. He also suggested that the new editor of the *Journal* should also be the permanent secretary, and this suggestion was adopted by the Executive Committee.

The treasurer's report showed that the finances of the association were in a most prosperous condition.

The annual addresses were delivered as follows: Medicine, Dr. J. C. Wilson, of Philadelphia; Surgery, Dr. F. W. McRae, of Atlanta; State Medicine, by Dr. Daniel Brower, of Chicago.

The Board of Trustees recommended that there should be no more a "permanent secretary," but that in the future the editor of the *Journal* should also act in this capacity, being provided with an assistant if necessary. This recommendation was adopted, and henceforth we will not see the beaming face of Dr. W. B. Atkinson, who for so long a time has filled the position of "permanent secretary."

The Nominating Committee made the following selection of officers for the ensuing year:

President, Dr. W. W. Keen, of Philadelphia; first vice-president, Dr. C. H. Wheaton, of St. Paul; second vice-president, Dr. E. D. Ferguson, of Troy, N. Y.; third vice-president, Dr. G. M. Allen, of Liberty, Mo.; fourth vice-president, Dr. W. E. D. Middleton, of Davenport, Iowa; secretary, Dr. Geo. H. Simmons, of Chicago; assistant-secretary, Dr. J. A. Joy, of Atlantic City, N. J.; treasurer, Dr. H. P. Newman, of Chicago.

The place chosen for the next meeting was Atlantic City, N. J.

---

THE daily press, soon after the meeting of the Georgia Medical Association in Macon, was filled with sensational items concerning a paper, or at least report, made by Dr. Stapler of Macon, before that body. Such sensational statements are to be deprecated as doing great harm to the furtherance of any scientific idea or to its correct interpretation by the laity. The reports were concerning the restoration of hearing and speech in part by an operation performed on a deaf mute. We had the pleasure of examining this patient through the courtesy of Dr. Stapler in his private office, and we feel that a few words would not be out of place. We fully believe that Dr. Stapler deplores such newspaper notoriety, for he is a well educated professional gentleman who has shown himself to be an original worker in the domain of otology and rhinology. What he has done has been in the way of scientific research, presenting his views first before a meeting of medical men, thus showing that he is performing a line of work which he hopes to be an advancement in the way of treating that *bête noire* of otology, deafness. The case in question happened to be one in which there were great possibilities of improvement, and he was fortunate enough to place himself in the hands of one who adopted the proper treatment, probably the first he had ever received. There are many cases of mutes where by the removal of certain pathological conditions both the speech and hearing can be brought out. This has been shown in an article by Dr. Frankenberger, of Prague, whose article we had the pleasure of translating two years ago. What was accomplished in this case might be duplicated in others, especially such as show the same pathological conditions—*i. e.*, adenoids and an impervious Eustachian tube. The method of electro-cauterization of the mouth of the Eustachian tube as advocated by Dr. Stapler, is exceedingly dangerous, and especially so when done without the aid of a mirror, as advocated by him. We think the doctor is over-enthusiastic, and this method could never commend itself to one who has done much rhinological and otological work.

---

THE Fourth Annual Meeting of the Association of Southern Railroad Surgeons was held in Richmond, Va., on May 29th and 30th. This association was organized in Atlanta four years ago, and since that time it has grown in interest at every succeeding meeting, both in point of membership and the excellent character of the papers presented. It is composed entirely of the surgeons who are connected with the Southern Railway system; and since this latter now embraces six thousand mileage in the States of Virginia, North and South Carolina, Georgia, Alabama, Mississippi, Tennessee and Kentucky, its membership is by no means insignificant. The meeting in Richmond was by far the best which has been held, and a great deal of its success must be attributed to the untiring zeal of that courtly and scholarly physician Dr. Geo. Ross, of that city. The program as presented, consisted of papers of the very highest order, showing that the author had given it the deepest study in its preparation. The members were all entertained at the home of Dr. Ross at an elegant informal "Reception Luncheon," which was purely a stag affair. Although the weather was warm, everything was done to make those present comfortable, and if appearances count for anything, the weather had no ill influence that day.

The association adjourned to meet in Charleston, S. C., on May 11th, 1900.

The following were the officers elected:

President, Dr. S. C. Dean, of Spartanburg, S. C.; first vice-president, Dr. W. C. Day, of Danville, Va.; second vice-president, Dr. Dunbar Roy, Atlanta; secretary and treasurer, Dr. T. H. Hancock, of Atlanta.

---

IT seems to us that there is very little aid extended to the medical profession through the existence and operation of the State Board of Medical Examiners. We were in hopes that through the active workings of such a board the state would not be so infested with the quasi-physicians and charlatan pretenders. The medical board is a good thing, and is an instrument for the production of great good, but the trouble is the offenders locate in the larger cities and there is no one to molest them as to the legality of their practice. They come in and settle down, and there is never any inquiry made as to whether they are practicing legally. Men of that character are slick dodgers, and are not adverse to trying to dodge the law while the new graduate in medicine and the conscientious doctor coming from another State would never think of trying to practice medicine without having first stood the state examination. The latter are caught, while the former, in the majority of instances, simply settle down and ply their trade. The officers of the law are lax in the discharge of their duty. We can go to-day and register our names in the book kept in the office of the Clerk of the Superior Court, pay our fifty cents, and these men who ought to see that the law is enforced, would never know whether or not we had passed the State board examination. The consequence of all this is that the reputable physician is made to stand his examination, while the quack and charlatan simply slide in and say nothing about it, knowing full well that his own standing will never be investigated. Something is radically wrong, and some action should be taken by which the Medical Examining Board of Georgia should be made effective.

---

WHILE we were unable to be present, we have since learned that the annual meeting of the American Medical Editors' Association, held in Columbus just before the meeting of the association proper, was in every way a success, and by no means without profit. The program as prearranged was in a large measure carried out. We quote from the *Journal of American Medical Association* some of the features of the program: "The session will be called to order at 2 P.M. in the hall of the Young Men's Christian Association building. The association, in addition, to routine business, will listen to a paper entitled 'The Ends and Aims of Medical Journalisms,' by Dr. J. D. Emmett, editor of the *American Gynecological and Obstetrical Journal.* In addition to this the committee have proposed a brief symposium upon the subject, "The Editor and the Author—the Rights of Each as Regards Contributed Articles." The committee has requested Dr. G. H. Simmons of the *Journal*, Dr. Riddle Goffe of the *Medical News*, Dr. Geo. M. Gould of the *Philadelphia Medical Journal*, Dr. J. C. Culberson of the *Cincinnati Lancet-Clinic*, and Dr. Dunbar Roy of the *Atlanta Journal-Record of Medicine*, to lead in the discussion. The annual dinner of the association is to be held at the Columbus Club at 6:30 P.M. Plates for the dinner $2 each."

---

THE Atlanta Board of Health has recently been making a great agitation and an endeavor to have all the wells in the city filled up so as to prevent the water therein from being used for drinking purposes. We think that this is a step in the right direction, and yet a step a little too far. We have had clinical experience to show that wells can hold the germs of typhoid fever, and can therefore be contaminated probably through the drains. Yet we do not think that such a sweeping law should be made as to require all the wells in the city to be filled. There are many poor people who are not able to keep their meats and butter in an ice refrigerator all the summer, but who are able to keep them in a bucket down in the well and derive all the benefit that a refrigerator could give, that probably never use the water for drinking purposes. Should the city deprive these people of such comforts which are inexpensive? Let the people be taught by card instructions sent to every home that well water, in many cases, is contaminated, and that for the benefit of their own household it should not be used. Don't fill up the wells, which are one of nature's refrigerators in summer and a balmy chimney in winter.

---

WE note that Dr. W. H. Peters of Lafayette, Ind., has gone into the advertisement business. We have just received a circular from the Peters Fountain Cuspidor Co., of which the doctor is president. He also sends out photographic views of his reception rooms and private office. We are not "art critics," but in the South we would be perfectly willing to place many of the physicians' offices in comparison, and even those which have never been photographed. "A thing of beauty is a joy forever." Selah!

